# Treatment outcomes and their predictors in children hospitalized with varicella complicated by bacterial superinfections after pandemic of COVID-19 – a retrospective multicenter analysis of real-life data in Poland

**DOI:** 10.1007/s10096-024-04944-2

**Published:** 2024-09-21

**Authors:** Maria Pokorska-Śpiewak, Leszek Szenborn, Maja Pietrzak, Magdalena Marczyńska, Anna Mania, Lidia Stopyra, Justyna Moppert, Kacper Toczyłowski, Artur Sulik, Filip Szenborn, Jolanta Jasonek, Inga Barańska-Nowicka, Adrianna Buciak, Ewa Majda-Stanisławska, Przemysław Ciechanowski, Katarzyna Karny, Ernest Kuchar, Magdalena Figlerowicz, Małgorzata Pawłowska

**Affiliations:** 1 Department of Children’s Infectious Diseases, Medical University of Warsaw, Warsaw, Poland; 2 Department of Pediatric Infectious Diseases, Regional Hospital of Infectious Diseases, Warsaw, Poland; 3 Department of Pediatrics and Infectious Diseases, Wroclaw Medical University, Wroclaw, Poland; 4 Department of Infectious Diseases and Child Neurology, Poznan University of Medical Sciences, Poznan, Poland; 5 Department of Infectious Diseases and Pediatrics, Department of Pediatrics, Żeromski Specialist Hospital in Krakow, Andrzej Frycz Modrzewski Krakow University, Krakow, Poland; 6 Department of Infectious Diseases and Hepatology, Faculty of Medicine, Collegium Medicum, Nicolaus Copernicus University, Bydgoszcz, Poland; 7 Department of Pediatric Infectious Diseases, Medical University of Bialystok, Bialystok, Poland; 8 Faculty of Information and Communication Technology, Wroclaw University of Science and Technology, Wroclaw, Poland; 9 Department of Pediatric Infectious Diseases, Medical University of Lodz, Lodz, Poland; 10 Department of Pediatrics and Infectious Diseases, Regional Hospital in Szczecin, Szczecin, Poland; 11 Department of Pediatrics with Clinical Assessment Unit, Medical University of Warsaw, Warsaw, Poland

**Keywords:** Antipyretics, Group a streptococcus (streptococcus pyogenes), Ibuprofen, Varicella

## Abstract

**Purpose:**

The aim of this study was to analyze treatment outcomes and their predictors in children hospitalized due to varicella complicated by bacterial superinfections after pandemic of COVID-19.

**Methods:**

This retrospective study analyzed data collected in a multicenter, nationwide, observational database dedicated for children aged 0–17 years hospitalized due to bacterial complications of varicella in 9 Polish tertiary healthcare inpatient centers. The primary endpoint of this study was the treatment outcome established after the end of hospital management assessed at a 4-point scale. The secondary endpoint was defined as the necessity of surgical intervention.

**Results:**

There were 458 patients with a median age of 4 (IQR 2–6) years. After the completed treatment, 319 (69%) participants were found fully recovered; 132 (29%) had transient complications; 2 (0.5%) had persistent complications; and 1 child (0.5%) died. Multivariate analysis revealed that implementation of ibuprofen in pre-treatment management of a child with varicella was associated with a 4.07-fold (2.50–6.60) increase in risk of complications after the treatment and it was associated with 2.87 times (1.39–5.89) higher risk of surgical intervention necessity. For other pre-hospital interventions (implementation of acyclovir, antibiotics or antihistaminics) no significant impact was observed. GAS infection increased the necessity of surgical intervention by 7.51 (3.64–15.49) times.

**Conclusions:**

One-third of patients treated for bacterial complications of varicella have post-treatment complications, most of them transient. GAS infection increases the need for surgical intervention. The use of ibuprofen in the treatment of varicella significantly increases the risk of complications and the need for surgical intervention.

## Introduction

Varicella (chickenpox) is a highly contagious disease caused by a primary infection with Varicella-Zoster virus (VZV) characterized by a vesicular rash frequently accompanied by fever and malaise [1]. It is common in childhood, and in most cases, its course is self-limiting. However, in some patients (2–3% or even over 15% according to the analyzed outpatient or inpatient cohort) it may be complicated, mainly by bacterial superinfection of skin and soft tissue, central nervous system symptoms or pneumonia [2, 3]. Bacterial infections are caused mainly by pathogens colonizing the skin, including group A β-haemolytic streptococci or Staphylococcus aureus, as varicella’s numerous skin lesions provide a portal entry to the deeper layers of the skin and underlying soft issue [1, 4]. Skin complications include several types of superinfection, including e.g., impetigo, cellulitis, ecthyma, scarlet fever, or abscess [3]. In addition, invasive infections like pneumonia, arthritis, osteomyelitis, necrotizing fasciitis, sepsis and septic shock may occur, and can be life threating [1]. Thus, despite the public perception of VZV infection as a harmless childhood disease, some patients require hospitalization due to potentially severe complications. On the other hand, varicella is a vaccine-preventable disease. The efficacy of a 2-dose schedule in preventing varicella is estimated at about 80–85% and more than 95% in the prevention of severe disease [1]. In countries, that implemented routine immunization against varicella, a significant decrease in its incidence was observed. In the USA, the universal vaccination program against varicella was introduced in 1995 and it resulted in a 90% reduction in morbidity and a 92% decline in complications in hospitalized patients [5–7]. However, as of 2021, less than half of European countries have introduced universal vaccinations against varicella [8]. In Poland, obligatory immunizations are available only for selected patients from risk groups, and do not cover the whole population. Thus, as most children remain susceptible for VZV infection, between 150,000 and 200,000 cases are reported annually, with the incidence rate between around 450–500/100,000 [9]. Approximately 0.5% of patients (around 1000 per year) require hospitalization [9]. The COVID-19 pandemic resulted in a significant decrease in varicella incidence, with 71,567 cases (incidence rate 186.6/100,000) in 2020 and 57,674 cases (150.37/100,000) in 2021 [9]. Since 2022, the number of cases has increased, returning to that reported in pre-pandemic years, with 171,708 cases (453.9/100,000) in 2022 and 190,715 (505.9100,000) in 2023, including 839 and 1084 patients requiring hospitalization, respectively [9].

Despite the fact that the risk of a severe course of varicella is significantly higher in certain risk groups (e.g., in the case of immunosuppression, pregnancy, newborns, and adulthood), most complications and hospital admissions occur in previously healthy children [1, 10–14]. Predictors of severe and complicated course of varicella in healthy, immunocompetent children are unclear. Thus, the aim of this study was to analyze treatment outcomes and their predictors in children hospitalized due to varicella complicated by bacterial superinfection after the pandemic of COVID-19.

## Methods

### Study design

This retrospective study analyzed data collected in a multicenter, nationwide, observational database dedicated to children hospitalized due to varicella (primary infection with varicella-zoster virus, VZV). In this database, we included epidemiological and clinical data on pediatric patients who have been hospitalized in 9 Polish tertiary healthcare inpatient centers, which reported their consecutive cases using an electronic questionnaire. Diagnosis of varicella was made by pediatric and infectious diseases specialists experienced in managing chickenpox, based on the typical clinical presentation. All pediatric patients reported in this database were managed and treated according to the current recommendations for children infected with VZV [15, 16]. Microbiological evaluation (including skin, blood, pharynx, conjunctiva, pus culture or rapid antigen testing for Streptococcus pyogenes) was performed according to the patient’s clinical presentation, based on the physicians recommendations. Skin swabs were obtained either from intact skin or from spontaneous or surgically obtained drainage of the lesion, if possible. This study included patients aged 0–17 years, diagnosed with varicella between July 1, 2022 and June 30, 2023, and hospitalized due to bacterial superinfection. The primary endpoint of this study was the treatment outcome established by the physician after the end of hospital management. It was assessed at a 4-point scale: 1 – healthy, fully recovered; 2 – with transient complications; 3 – with persistent complications; and 4 – death. The secondary endpoint was defined as the necessity of surgical intervention.

Epidemiological data included age, sex, immunosuppression, the putative source of VZV infection, and the number of days from disease onset to admission to the hospital. In addition, the following clinical data were collected: the pre-hospital management (use of acyclovir, antibiotics, antipyretics including ibuprofen, antipruritics), clinical presentation of superinfection, laboratory and microbiological evaluation, treatment and its outcomes.

### Statistical analysis

Categorical data were presented as numbers with percentages and were compared using the chi-square test. Continuous variables were presented as medians (interquartile ranges, IQR). Regression analyses were conducted to identify predictors of treatment outcomes in the study group. Two separate models were constructed: one analyzing predictors of treatment outcome assessed at other than ‘1 – healthy, fully recovered” on a 4-point scale and the second, aiming to determine factors associated with the necessity of surgical intervention. For both models, a multiple regression was performed with the following variables (candidate predictors) entered into the model irrespective of the results of the univariate analysis: age, sex, source of infection (household vs. other), implementation of acyclovir, antibiotics, ibuprofen or antipruritics before hospitalization, infection with *Streptococcus pyogenes*. After entering all the variables into the model, those variables that showed the least significant associations were subsequently excluded until all variables remained significant (p < 0.05). The results were presented as odds ratios (ORs) and 95% confidence intervals (95%CI). Results with CI not including 1.0 were considered statistically significant. All statistical analyses were performed using MedCalc Statistical Software version 22.018 (MedCalc, Ostend, Belgium, https://www.medcalc.org). A two-sided p-value of < 0.05 was considered significant.

### Ethical statement

As the study was retrospective, non-interventional, and based on data collected anonymously in the national database, ethical review and approval were waived. Due to the retrospective nature of the presented study, written consent was not obtained from participants. The patients’ data were protected according to the European Union General Data Protection Regulation.

## Results

### Participants

There were 458 patients included in the study group (244 boys and 214 girls) with a median age of 4 (IQR 2–6) years. Only 6/458 were immunocompromised. The source of infection was established for 52% of patients, with household contact dominating (30%). Most children were still infectious at admission to the hospital, which occurred at a median of 4 (3–5) days after the onset of the disease. No child in the studied group had received a full 2-dose course of vaccination against varicella, and one patient was reported to had been vaccinated with one dose. The baseline characteristics of the study group were presented in Table 1.

**Table 1:** Baseline characteristics of 458 patients in the study group

Characteristics		Number (%)/Median (IQR)
Sex	Male/Female	244 (53%)/ 214 (47%)
Age	Median of years (IQR)	4 (2–6)
	< 12 months	55 (12%)
	1–5 years	265 (58%)
	6–12 years	122 (27%)
	> 12 years	16 (3%)
Immunosuppression	Yes	6 (1%)
Source of VZV infection	Household	137 (30%)
	Daycare	94 (21%)
	Other (e.g., refugee camp, friends)	6 (1%)
	Not established	221 (48%)
Child being Infectious at admission	Yes	409 (89%)
Day of varicella at admission	Median of days (IQR)	4 (3–5)
	Days (range)	1–15
Day of varicella when complications occurred	Median of days (IQR)	3 (2–4)
	Days (range)	1–14

VZV – varicella-zoster virus, IQR – interquartile range

### Pre-hospital management

Half of the study participants (231/458) received antiviral treatment with acyclovir before admission. Antibiotics were implemented in 40 (9%) patients, including amoxicillin (with or without clavulanic acid) in 18 children, and cephalosporins (first or second generation) in 16 patients. Antipyretics were used in 368 (80%) of children: among them 266 (72%) received paracetamol, 111 (30%) ibuprofen, and 22 (6%) metamizole. In 110 (30%) participants two of these drugs were prescribed (including paracetamol plus ibuprofen in 95, 26%; and paracetamol plus metamizole in the remaining 15, 4%), and three drugs in 2 (0.5%) of children. Antihistaminic drugs as antipruritics were used in 107 (23%) of patients.

### Hospital management

**Fig. 1:**
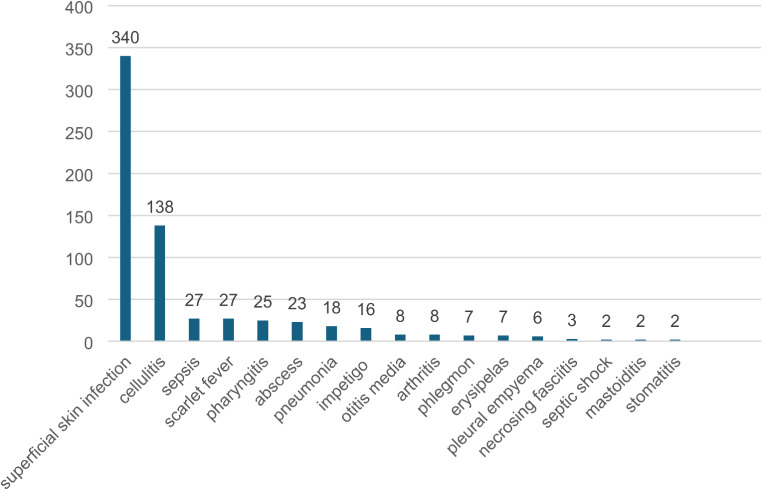
Number of patients presenting with different types of bacterial superinfections. In 117 (26%) of patients, more than one type of complication was diagnosed

Superficial bacterial superinfections of the skin were most common and they were diagnosed in 340 participants. Other skin and soft tissue complications included cellulitis, scarlet fever, abscess, impetigo, phlegmon, erysipelas, and necrotizing fasciitis (Fig. 1). In 27 (6%) patients sepsis was diagnosed, and septic shock in 2 children (one with confirmed GAS etiology, and one caused by Streptococcus constellatus and Prevotella timonensis). Head and trunk were among the most affected areas, and in 163 (36%) patients, more than one skin area was involved. Among non-skin bacterial complications of varicella, there were: pharyngitis, pneumonia, otitis media, arthritis, pleural empyema, mastoiditis, and stomatitis (Fig. 1). In 117 (26%) of patients, more than one type of complication was diagnosed.

Microbiological evaluation revealed the etiology of superinfection in 246 (54%) of children, with two dominant bacteria: group A Streptococcus (GAS, Streptococcus pyogenes) and Staphyloccocus aureus (Table 2).

**Table 2:** Microbiological evaluation

Etiology	Skin swab	Blood culture	Other (pharynx, conjunctiva, pus)	Overall number of patients with this etiology
*Streptococcus pyogenes*	120 (26%)	22 (5%)	18 (4%)	142 (31%)
*Staphylococcus aureus* MSSA	72 (16%)	3 (0.5%)	1 (0.5%)	74 (16%)
*Staphylococcus aureus* MRSA	2 (0.5%)	-	-	2 (0.5%)
*Streptococcus pyogenes + Staphylococcus aureus*	14 (3%)	1 (0.5%)	-	14 (3%)
Other	10 (2%)	4 (1%)	-	14 (3%)
No growth	26 (6%)	39 (9%)	-	-
Etiology established (overall)	208 (45.5%)	30 (7%)	19 (4.5%)	246 (54%)

MRSA – methicillin-resistant Staphylococcus aureus; MSSA- methicillin-sensitive Staphylococcus aureus

All children received antibiotics, mainly beta-lactams (cephalosporins, amoxicillin, penicillin). In 99 (22%) participants, combined antibiotic therapy (beta-lactams with clindamycin or vancomycin) was used. The median duration of antibiotic therapy was 10 (7–10) days, and the median duration of hospitalization was 5 (4–8) days, ranging from 1 to 26 days. Surgical intervention was necessary in 44 (10%) of patients, including 26 (6%) who were transferred to the surgical ward. Four children (1%) required treatment in the intensive care unit (ICU).

### Treatment outcomes and their predictors

After the completed treatment, 319 (69%) participants were found to be healthy, fully recovered; 132 (29%) had transient complications; 2 (0.5%) had persistent complications; and 1 child (0.5%) died. For 4 (1%) patients data was not available. One death occurred in a 4-year-old girl with newly diagnosed leukemia due to the multi-organ failure; this child, however, was also diagnosed with a septic shock caused by Streptococcus constellatus and Prevotella timonensis.

Patients’ status after the treatment was not related to their age, as persistent or transient complications occurred in 27% of infants < 12 months, 33% of children aged 1–5 years, and 26% of patients > 5 years of age (*p* = 0.65). The analysis of the clinical status of patients according to pre-hospital management and GAS-etiology of superinfection revealed that children treated with ibuprofen significantly more frequently presented with complications after the treatment compared to patients who had not received this antipyretic (50% vs. 22.5%; *p* < 0.0001). Other pre-hospital interventions or GAS infections did not lead to significant differences in treatment outcomes.

Multivariate analysis revealed that implementation of ibuprofen in pre-treatment management of a child with varicella was associated with a 4.07-fold (2.50–6.60) increase in the risk of complications after the treatment of bacterial superinfection (Table 3A). In addition, it was associated with a 2.87-fold (1.39–5.89) increased risk of surgical intervention necessity (Table 3B). For other pre-hospital interventions, significant impact was not observed. However, GAS infection also increased the necessity of surgical intervention 7.51 (3.64–15.49) times (Table 3B).

**Table 3:** Predictors of treatment outcomes in children hospitalized due to varicella complicated by bacterial superinfections (multivariate analysis)

Predictor	Odds ratio (95% CI)
**A. Predictors of complications after the treatment**	
Age (years)	0.96 (0.90–1.02)
Sex (Male vs. female)	1.24 (0.80–1.92)
Source of VZV infection (household vs. other)	0.62 (0.37–1.02)
Use of acyclovir in pre-hospital management	1.44 (0.92–2.24)
Use of antibiotic in pre-hospital management	1.46 (0.70–3.02)
Use of ibuprofen in pre-hospital management	**4.07 (2.50–6.60)**
Use of antipruritics in pre-hospital management	0.87 (0.74–1.03)
GAS infection	0.65 (0.37–1.02)
**B. Predictors of surgical intervention necessity**	
Age (years)	0.96 (0.85–1.08)
Sex (Male vs. female)	0.93 (0.47–1.83)
Source of VZV infection (household vs. other)	0.53 (0.22–1.27)
Use of acyclovir in pre-hospital management	1.30 (0.65–2.59)
Use of antibiotic in pre-hospital management	0.85 (0.25–2.87)
Use of ibuprofen in pre-hospital management	**2.87 (1.39–5.89)**
Use of antipruritics in pre-hospital management	0.95 (0.73–1.24)
GAS infection	**7.51 (3.64–15.49)**

GAS – group A Streptococcus (Streptococcus pyogenes); VZV – varicella-zoster virus

## Discussion

In this study, we aimed to analyze treatment outcomes in children with varicella complicated by bacterial superinfections in a one-year period after the COVID-19 pandemic. During the analyzed period, 207,409 cases of varicella were reported in Poland, which is almost 15% more than in the pre-pandemic year 2019, in which 180,641 cases were noted with an incidence rate of 0.64/100,000 [9]. Although in 2022 and 2023 the proportion of hospitalized patients was even lower than in 2019 (0.49% and 0.57% compared to 0.64%, respectively), there was a 2.66-fold increase in the number of children hospitalized in our 9 main Polish pediatric infectious disease centers due to varicella complicated by bacterial superinfections from 172 in 2019 to 458 in the analyzed period. This cannot be explained by the 15% increase in number of patients suffering from varicella after COVID-19 pandemic. Due to the control measures (e.g., lockdown) during the pandemic, immunity gaps were expected to provoke a graver progression of the illness or an increased risk of acquiring more than one infectious disease simultaneously. In addition, the most commonly confirmed bacterial pathogen in our cohort was GAS, whose prevalence increased significantly across Europe after the pandemic [17]. In a study from the Netherlands, a 7-fold rise of invasive GAS infections was observed after the pandemic compared to pre-pandemic years [18]. In Poland, there was an over 2-fold increase in the number of scarlet fever in 2023 compared to 2019 (44,644 vs. 20,838, respectively). The prevalence of invasive GAS infections did not alter significantly (6225 vs. 6489, respectively), however, a 5-fold increase in toxic shock syndrome cases was observed in 2023 compared to 2019 (100 vs. 21 cases) [9]. It has been demonstrated that varicella itself is a leading predisposing factor for GAS infections, and utilization of varicella vaccine leads to a significant decline in hospitalizations due to varicella-associated GAS infection [19]. GAS is considered the most common bacterial cause of varicella coinfections [20], and usually the superinfection occurs as the result of infected skin lesions, which provide portals for bacterial entry into the blood [21]. Both epidemiological and clinical reports support the idea that not only VZV, but several other viruses including influenza, Epstein-Barr, and morbilli virus increase the incidence of GAS complications [21]. Molecular bases underlying these observations are limited, however, virus-induced changes in bacterial adherence and effector cell-mediated clearance appear to be responsible for this phenomenon [21, 22]. In addition, coinfection of viral diseases with different bacteria (e.g., GAS) or other viruses may complicate the differential diagnosis and can lead to missing or delayed identification of the proper etiology [23]. In these cases, molecular testing for GAS and VZV might be helpful. The addition of new molecular assays and next-generation sequencing has broadened diagnostic capabilities for identifying infectious agents [24]. Currently, polymerase chain reaction (PCR) and direct fluorescence assays (DFA) are considered the methods of choice for VZV infection confirmation, with PCR (including quantitative PCR) considered the most sensitive and reliable testing method for VZV to date [25]. In addition, a new generation of molecular GAS rapid tests is now available with higher sensitivity than rapid antigen detection tests [26].

A recent retrospective study that analyzed a cohort of 221 children hospitalized due to varicella before and after the COVID-19 pandemic found that the patients with primary VZV infection admitted in 2022 presented with a more severe course of the disease compared to those from 2019. The risk of bacterial superinfection was 3.38 (95% CI 1.80–6.35) times higher, with sepsis occurring 5.70 (1.31–24.77) times more frequent in 2022 than in pre-pandemic year 2019 [27].

Most cases of varicella are mild, however an estimated 2–6% of cases are complicated [28]. A recent cohort analysis of real data from France on 48,027 patients with varicella in outpatient settings revealed that even 15.3% of them (7369) presented with complications, and 17–25% required antibiotic treatment (2). In this cohort, complications were most frequent among infants (19.2%) and adolescents aged 13–18 (18.1%). Interestingly, in most studies, complications occur among otherwise healthy children, which is concordant with our cohort, in which immunosuppressed children constituted only 1% of the study group [1, 3, 14]. Bacterial skin infections are usually considered as the most frequent complications of varicella [3, 12, 14, 19, 29]. According to the recent systematic review, skin-related complications account for 20.12% of all varicella-related complications and they are more prevalent in children compared to adults [29]. In a Brazilian study analyzing complications of VZV infection among 669 hospitalized children, bacterial complications were the most common cause of admission (77.7%) [14]. In this cohort, 44 patients (6.6%) required admission to the ICU and 5 (0.8%) died of septic shock. In our group numbers of both ICU admissions (4) and deaths (1) were significantly lower. However, one third of our cohort presented with complications (mainly transient) after the treatment. It has been shown that patients infected through the household contact with VZV may be at risk for unfavorable outcomes and complications [14]. This hypothesis was not confirmed by our study, as source of the infection was not revealed as predictor of treatment outcomes.

Other factors considered as predictors of bacterial complications include the use of nonsteroidal anti-inflammatory drugs (NSAIDs), recurrence of fever, age < 1 year, and thrombocytopenia [3, 12, 14]. In our study, the use of ibuprofen was associated with worse treatment outcomes, increased risk of complications and necessity of surgical intervention. So far, treatment with ibuprofen has been considered controversial, as some data suggested its association with life-threatening streptococcal skin infections, cellulitis, abscess, and necrotizing soft tissue infections in patients (mainly children) with varicella [3, 4, 30–33]. In addition, NSAIDs are not only able to promote the development of bacterial superinfections, but they may also mask symptoms and cause a delay in the proper management [30]. However, several prospective studies have given conflicting results in this field. Thus, this association cannot be confirmed or ruled out with certainty [4, 31]. A study by Mikaeloff et al. conducted on 140,111 individuals with chickenpox during a 12-year observation period confirmed that the use of NSAIDs was associated with an increased risk of skin complications [32]. On the other hand, Lesko et al. analyzing 224 patients with varicella, found that the use of ibuprofen was not associated with a higher risk of soft tissue necrosis, but the probability of GAS infection was 3.9-fold higher in patients taking ibuprofen [4]. In our study, both these factors (the use of ibuprofen and GAS infection) were associated with an increased risk of necessity for surgical intervention. In addition, our study was not designed to find an association between ibuprofen and varicella complications, but to find predictors of the treatment outcomes. Thus, to the best of our knowledge, this is the first analysis demonstrating the influence of ibuprofen use on the outcomes in children treated for bacterial complications of varicella. However, it should also be taken into consideration that ibuprofen, and in particular ibuprofen with paracetamol, may be used to treat more severely ill children. Thus, the severity of the predisposing varicella may be a risk factor for subsequent invasive GAS infection and not only the implemented antipyretic drug [4].

Other analyzed factors (pre-hospital implementation of acyclovir, antibiotics, or antipruritics) were not associated with prognosis. It was demonstrated that using acyclovir within 24 h of illness resulted in a 1-day reduction of fever and 15–30% reduction in the severity of signs and symptoms [34]. However, acyclovir treatment does not reduce the rate of complications [1], which is concordant with our results. Secondary bacterial infection in the course of varicella requires rapid implementation of antibiotic therapy. According to the French real-world data, even 25.1% of over 48,000 patients with varicella in the outpatient setting were prescribed antibiotics, including 68.1% of patients with complications, and 17.3% uncomplicated, which contributed to the 23% of total varicella-related costs annually [2]. Among the 3 most commonly prescribed medications in this French cohort, there were also antihistamines (74.1%, significantly more frequent compared to our group), and analgesics (69.9%).

Our multicenter study was performed on a large group of patients with bacterial complications of varicella during a 12-month post-COVID observation period in a country without a universal vaccination program. However, several limitations of this study should be noted. The first issue is its retrospective nature, which did not allow causes and effects to be distinguished. Second, there could be referral bias, as only tertiary referral centers reported their data. However, due to high infectivity of varicella, general pediatric departments usually refuse to admit such patients. In addition, treatment outcome was assessed by a physician subjectively, without detailed data on the type of complications. Moreover, we cannot rule out that ibuprofen was implemented in more severely ill children at baseline, and that the severity of the predisposing varicella could also pose a risk for worse outcomes.

In conclusion, the results of this multicenter study performed on a large cohort of hospitalized children revealed that one-third of patients treated for bacterial complications of varicella have post-treatment complications, most of them transient. GAS infection in a patient with varicella leads to the need for surgical intervention significantly more often than other etiologies of superinfection. The use of ibuprofen in the treatment of varicella significantly increases the risk of complications and the need for surgical intervention. Therefore, this drug should not be used in children suffering from varicella.

## Data Availability

The datasets used and analyzed during the current study are available from the corresponding author upon reasonable request.
